# Neuroinflammation and oxidative stress in rostral ventrolateral medulla contribute to neurogenic hypertension induced by systemic inflammation

**DOI:** 10.1186/1742-2094-9-212

**Published:** 2012-09-07

**Authors:** Kay LH Wu, Samuel HH Chan, Julie YH Chan

**Affiliations:** 1 Center for Translational Research in Biomedical Sciences, Chang Gung Memorial Hospital-Kaohsiung Medical Center, Kaohsiung, 83301, Taiwan

**Keywords:** Neuroinflammation, Pro-inflammatory cytokines, Microglia activation, Cycloxygnase-2, Oxidative stress, Kv4.3 channel, Hypertension

## Abstract

**Background:**

In addition to systemic inflammation, neuroinflammation in the brain, which enhances sympathetic drive, plays a significant role in cardiovascular diseases, including hypertension. Oxidative stress in rostral ventrolateral medulla (RVLM) that augments sympathetic outflow to blood vessels is involved in neural mechanism of hypertension. We investigated whether neuroinflammation and oxidative stress in RVLM contribute to hypertension following chronic systemic inflammation.

**Methods:**

In normotensive Sprague-Dawley rats, systemic inflammation was induced by infusion of *Escherichia coli* lipopolysaccharide (LPS) into the peritoneal cavity via an osmotic minipump. Systemic arterial pressure and heart rate were measured under conscious conditions by the non-invasive tail-cuff method. The level of the inflammatory markers in plasma or RVLM was analyzed by ELISA. Protein expression was evaluated by Western blot or immunohistochemistry. Tissue level of superoxide anion (O_2_^·-^) in RVLM was determined using the oxidation-sensitive fluorescent probe dihydroethidium. Pharmacological agents were delivered either via infusion into the cisterna magna with an osmotic minipump or microinjection bilaterally into RVLM.

**Results:**

Intraperitoneal infusion of LPS (1.2 mg/kg/day) for 14 days promoted sustained hypertension and induced a significant increase in plasma level of C-reactive protein, tumor necrosis factor-α (TNF-α), or interleukin-1β (IL-1β). This LPS-induced systemic inflammation was accompanied by activation of microglia, augmentation of IL-1β, IL-6, or TNF-α protein expression, and O_2_^·-^ production in RVLM, all of which were blunted by intracisternal infusion of a cycloxygenase-2 (COX-2) inhibitor, NS398; an inhibitor of microglial activation, minocycline; or a cytokine synthesis inhibitor, pentoxifylline. Neuroinflammation in RVLM was also associated with a COX-2-dependent downregulation of endothelial nitric oxide synthase and an upregulation of intercellular adhesion molecule-1. Finally, the LPS-promoted long-term pressor response and the reduction in expression of voltage-gated potassium channel, Kv4.3 in RVLM were antagonized by minocycline, NS398, pentoxifylline, or a superoxide dismutase mimetic, tempol, either infused into cisterna magna or microinjected bilaterally into RVLM. The same treatments, on the other hand, were ineffective against LPS-induced systemic inflammation.

**Conclusion:**

These results suggest that systemic inflammation activates microglia in RVLM to induce COX-2-dependent neuroinflammation that leads to an increase in O_2_^·-^ production. The resultant oxidative stress in RVLM in turn mediates neurogenic hypertension.

## Background

There are increasing indications that chronic inflammation plays a critical role in the pathogenesis of cardiovascular diseases, including atherosclerosis, heart failure, diabetes mellitus, and hypertension [1-4]. In addition to the well characterized involvement of proinflammatory cytokines, including tumor necrosis factor-α (TNF-α) and interleukins (ILs) in endothelial cells [5], and vascular smooth muscle cells [5,6] in cardiovascular remodeling during disease conditions, recent focus has shifted to the role of brain inflammation in the pathogenesis of cardiovascular diseases [2,4,7,8]. For example, expression of both TNF-α and IL-1β in the paraventricular nucleus of hypothalamus (PVN), a cardiovascular regulatory region in the forebrain, is increased in rats with ischemia-induced heart failure [9]. Activation of the peripheral or brain renin-angiotensin system increases the production of pro-inflammatory cytokines within specific brain regions involved in blood pressure control [7,8,10], resulting in hypertension. In the spontaneously hypertensive rats, the pro-inflammatory chemotactic proteins are highly expressed in brain stem nuclei involved in cardiovascular regulation [11,12]. Treatments that are beneficial to heart failure [9] or hypertension [13,14] also promote anti-inflammatory effects in the brain.

Neuroinflammation is associated with an increase in sympathetic drive during cardiovascular disease [2,15]. An increase in sympathetic outflow to the peripheral vasculature from the rostral ventrolateral medulla (RVLM), where premotor neurons for the maintenance of sympathetic vasomotor activity are located [16], contributes to neural mechanism of hypertension [17]. Abundant evidence now suggests that oxidative stress because of an imbalance of production over degradation of the reactive oxygen species (ROS), in particular superoxide anion (O_2_^·-^), in RVLM contributes to neurogenic hypertension [18,19]. Whether neuroinflammation serves as a molecular signal for ROS activation in RVLM, leading to neurogenic hypertension, however, is currently unknown.

A well-studied model of peripheral inflammation in rodents is systemic administration of *Escherichia coli* lipopolysaccharide (LPS) from gram-negative bacteria to induce innate immune response. The present study took advantage of this model to evaluate changes in the expression of pro-inflammatory cytokines in RVLM during the course of chronic systemic inflammation and delineate the underlying mechanisms. We also deciphered the role of ROS in RVLM on development of neurogenic hypertension following peripheral LPS administration. Our data show that long-term LPS-induced systemic inflammation activates microglia and increases the expression of proinflammatory cytokines in RVLM, leading to oxidative stress-associated neurogenic hypertension.

## Methods

### Animals

Adult, male Sprague-Dawley rats (10-week-old, 200 to 250 g, *n*=146) were purchased from the Experimental Animal Center of the National Applied Research Laboratories, Taiwan. Animals were maintained under temperature control (24±0.5°C) and 12-h light-dark cycle (lights on between 08:00 and 20:00), and provided with standard chow and tap water *ad libitum*. All experimental procedures were carried out in compliance with the guidelines of our institutional animal care and use committee.

### Induction of low-grade systemic inflammation

Systemic inflammation was induced by continuous infusion of *Escherichia coli* LPS into the peritoneal cavity for 14 days via an osmotic minipump. On the day of implantation, animals were anesthetized with sodium pentobarbital (50 mg/kg, i.p.) and an osmotic minipump (Alzet 1002; Durect Co., Cupertino, CA, USA) was placed in the peritoneal cavity. Control animals received saline-filled osmotic minipumps, and sham-operated animals received identical surgical procedures only. We found in our pilot study that whereas low doses of LPS (0.3 to 1.2 mg/kg/day) infusion induced systemic inflammation and a long-term pressor response, higher dose (5.0 or 10 mg/kg/day) resulted in fever and septic-like hypotension. Intraperitoneal (IP) infusion of LPS at a dose of 1.2 mg/kg/day was therefore used in the present study to induce low-grade systemic inflammation.

### General experimental protocol

Baseline systemic arterial pressure (SAP) and heart rate (HR) were recorded for 3 days, followed by daily recording after IP infusion of LPS (1.2 mg/kg/day) or saline for 14 days. Body weight, body temperature, food, and water intake of the animals were recorded daily for 14 days. Some animals received additional intracisternal (IC) or IP infusion of NS398 (1.5 nmol/μL/h), minocycline (9 nmol/μL/h), PTX (30 nmol/μL/h), tempol (1 μmol/μL/h), or aCSF for 14 days; or additional IP infusion of the same dose of NS398, minocycline, or tempol for 14 days. Other groups received additional microinjection bilaterally into RVLM or intravenous injection of the same dose of tempol on day 7 after the beginning of LPS infusion. At various post-treatment intervals, animals were killed to collect blood and RVLM tissues for molecular, biochemical, and immunohistochemical experiments.

### Measurement of systemic arterial pressure and heart rate

We routinely measured SAP and HR between 14:00 and 16:00 in conscious rats using the non-invasive tail-cuff electrosphygmomanometry (MK-2000; Momuroki Kikai Co., Japan) method according to the previously described procedures [20]. Tail-cuff plethysmography instead of radiotelemetry was used in this study for measurement of SAP to reduce potential confounding influence by the implanted telemetry transmitter in the peritoneal cavity on the induced systemic inflammation. We have previously validated that SAP and HR obtained by tail-cuff plethysmography were comparable to those measured by radiotelemetry [20].

### Power spectral analysis of systemic arterial pressure signals

The recorded SAP signals were simultaneously subject to power spectral analysis. We were particularly interested in the low-frequency (LF, 0.25 to 0.8 Hz) components of SAP signals. This spectral component of SAP signals was reported to take origin from the RVLM [21], and reflect the prevalence of baroreflex-mediated sympathetic neurogenic vasomotor tone [22].

### Analysis of plasma inflammatory markers

One milliliter of blood was collected from the heart and mixed with 1 mL Minicollect® tripotassium EDTA (Greiner bio-one, Monroe, NC, USA) for quantitative analysis of TNF-α, IL-1β, or IL-6 by the sandwich ELISA (Bender MedSystems, Burlingame, CA, USA) according to the manufacturer’s instructions. Plasma C-reactive protein (CRP) was measured by BD™ ELISA Rat CRP kit (BD Biosciences, San Diego, CA, USA). The colorimetric reaction product for individual pro-inflammatory factor was measured at 450 nm using a microplate reader (Dynex, Chantilly, VA, USA). The concentration of TNF-α, IL-1β, IL-6, or CRP, expressed in micrograms per milliliter (μg/mL), was determined from the regression line for the standards incubated under the same conditions in each assay. All assays were performed in triplicate.

### Collection of tissue samples from RVLM

At various time intervals after experimental treatment, rats were killed with an overdose of pentobarbital sodium (100 mg/kg, i.p.) and perfused intracardially with warm saline. The brain was rapidly removed and immediately frozen on dry ice. The medulla oblongata covering RVLM was blocked between 0.5 and 1.5 mm rostral to the obex, as adopted from the atlas of Watson and Paxinos [23]. Both sides of the ventrolateral medulla covering RVLM (approximately 1.5-mm to 2.5-mm lateral to the midline and medial to the spinal trigeminal tract) were collected by micropunches with a 1-mm inner diameter stainless-steel burr [18-20]. Medullary tissues collected were stored at -80°C for subsequent protein analysis.

### Total protein isolation

Tissue samples from RVLM were homogenized with a Dounce grinder with a tight pestle in ice-cold lysis buffer (15 mM HEPES, pH 7.2, 60 mM KCl, 10 mM NaCl, 15 mM MgCl_2,_ 250 mM Sucrose, 1 mM EGTA, 5 mM EDTA, 1 mM PMSF, 2 mM NaF, 4 mM Na_3_VO_4_,). A mixture of leupeptin (8 μg/mL), aprotinin (10 μg/mL), phenylmethylsulfonyl fluoride (20 μg/mL), and trypsin inhibitor (10 μg/mL) was included in the isolation buffer to prevent protein degradation. The homogenate was centrifuged at 13,500*g* for 10 min, and the supernatant was collected for protein analysis. The concentration of the total protein extracted was estimated by the method of Bradford with a protein assay kit (Bio-Rad, Hercules, CA, USA).

### Analysis of inflammatory markers in RVLM

Total protein extracted from RVLM was subject to quantitative analysis of TNF-α, IL-1β, IL-6, or prostaglandin E2 (PGE2) by the sandwich ELISA (Bender MedSystems, Burlingame, CA, USA) according to the procedures described above. The concentration of each proinflammatory factor was expressed in picograms per milliliter (pg/mL). All assays were performed in triplicate.

### Western blot analysis

Total protein extracted from RVLM samples was subject to Western blot analysis according to the procedures described previously [20,22,24]. The primary antisera used included goat polyclonal antiserum against p47^phox^ (1:5,000; Santa Cruz, CA, USA), rabbit polyclonal antiserum against Iba-1 (1:1,000; Wako, Tokyo, Japan), catalase (1:4,000; Stressgen, Ann Arbor, MI, USA), copper/zinc SOD (Cu/ZnSOD, 1:3,000; Stressgen), manganese SOD (MnSOD, 1:6,000; Stressgen), extracellular SOD (ecSOD, 1:5,000; Stressgen), goat polyclonal antiserum against Kv4.3 (1:1,000; Santa Cruz), or mouse monoclonal antiserum against glutathione peroxidase (GPx, 1:5,000; BD Biosciences), gp91^phox^ (1:5,000; BD Biosciences), endothelial NOS (eNOS, 1:5,000; BD Biosciences), neuronal nitric oxide synthase (nNOS, 1:5,000; BD Biosciences), inducible NOS (iNOS, 1:5,000; BD Biosciences), COX-2 (1:1,000; Cayman, Ann Arbor, MI, USA), or intercellular adhesion molecule-1 (ICAM-1) (1:5,000; Abcam, Cambridge, MA, USA). Membranes were washed with TBS-t buffer followed by the secondary antibodies (1:10,000; Jackson ImmunoResearch, West Grove, PA, USA). This was followed by incubation with horseradish peroxidase-conjugated goat anti-rabbit IgG or goat anti-mouse IgG (Jackson ImmunoReserach). Specific antibody-antigen complex was detected using an enhanced chemiluminescence Western blot detection system (GE Healthcare Bio-Sciences Corp., Piscataway, NJ, USA). The amount of detected protein was quantified by the Photo-Print Plus software (ETS Vilber-Lourmat, Marne-la-Vallée, France), and was expressed as the ratio to β-actin protein.

### Measurement of superoxide anion

To measure the tissue level of O_2_^·-^, the extracted proteins (20 μg/test) from RVLM were incubated with the oxidation-sensitive fluorescent probe dihydroethidium (DHE, 1 μM; Invitrogen, Carlsbad, CA, USA). After 15 min of incubation under protection from light, the suspension was subject to fluorescence analysis (FluorStar; Biodirect, Inc., Taunton, MA, USA). All measurements were performed in triplicate. For *in situ* identification of O_2_^·-^ in RVLM, rats were killed with an overdose of pentobarbital sodium (100 mg/kg, i.p.) and perfused intracardially with warm saline. The brain stem was rapidly removed, mounted in OCT compound (Leica Microsystems, Nussloch, Germany), and frozen at -20°C. A series of coronal sections (at 35 μm) through the RVLM (0.5 to 1.5 mm rostral to the obex) were cut using a cryostat (Leica, Houston, TX, USA). These sections were incubated for 30 min in the dark with DHE (1 μM). After washing with phosphate buffered saline (PBS), DHE fluorescence was visualized by fluorescence microscopy (Olympus Optical, Tokyo, Japan) using an excitation wavelength of 543 nm and a rhodamine emission filter [20,25].

### Immunohistochemistry and immunofluorescence

Animals were perfused transcardially with 4% paraformaldehyde in 0.1 M PBS (pH 7.4) under deep pentobarbital anesthesia (100 mg/kg, i.p.), and the brain stem was removed and post-fixed overnight in the same fixative, followed by 30% sucrose solution for at least 3 days. Coronal sections of the rostral medulla oblongata at 35 μm were cut using a cryostat (Leica). After pre-absorption in gelatin (0.375%), normal horse serum (3%), and triton-X 100 (0.2%) in PBS, the sections were incubated with a rabbit polyclonal antibody against Iba-1 (1:1,000; Wako), at room temperature overnight and then rinsed in PBS. After incubation in biotinylated horse anti-rabbit IgG (1:200; Jackson ImmunoResearch, the sections were rinsed in PBS and incubated with AB complexes (Vectastain ABC elite kit, Vector Laboratories, Burlingame, CA, USA). This was followed by washing the sections in PBS and incubated with a 3,3′-diaminobenzidine substrate kit (Vector Laboratories). Sections were counter stained with 1% Neural Red, and observed under a light microscope (Olympus) [26].

For immunofluorescence experiments, a mouse monoclonal antiserum against ICAM-1 (1:1,000, Biovision) and a rabbit polyclonal antiserum against a blood glycoprotein Von Willebrand factor (vWF; 1:1,000, Abcam) were incubated at room temperature overnight. After incubation in Alex 568 conjugated anti-mouse IgG (1:200; Invitrogene) and Alex 488 conjugated anti-rabbit IgG (1:200; Jackson Invitrogene), the sections were rinsed in distilled water. Sections were mounted and observed under a laser confocal microscope (FluoView FV10i, Olympus) [20,25].

### Quantitative evaluation of Evans blue extravasation

Blood-brain barrier disruption was assessed quantitatively by measuring Evans blue (EB; Sigma-Aldrich, St Louis, MO, USA) extravasation [27]. Briefly, rats were injected intravenously with 0.1 mL 2% EB, and the dye was allowed to circulate for 60 min. The animals were perfused transcardially with 0.9% PBS to remove intravascular EB dye. Tissue sample from RVLM was weighed and homogenized in 500 μL 0.1 M PBS and 500 μL of pure trichloroacetic acid (Sigma-Aldrich). Samples were incubated at 4°C for at least 1 h and centrifuged at 10,000 g for 30 min. The resultant supernatants were measured for absorbance of EB at 610 nm using a spectrophotometer (Thermo Fisher Scientific, Waltham, MA, USA). All measurements were performed in triplicate.

### Intracisternal infusion of test agents

Intracisternal (IC) infusion of test agents was carried out according to the previously described procedures [20,25]. In brief, after the dura mater between the foramen magnum and C1 lamina was perforated with a 22-gauge steel needle, a PE-10 catheter (Clay Adams, Sparks, MD, USA) was advanced for 5 mm into the cisterna magna. The catheter was sealed to the dura with tissue glue and the incision was closed with layered sutures. The outer end of the catheter was connected to an osmotic minipump (Alzet 1002), which was placed under the skin in the neck region [20,25]. IC infusion of a cyclooxygenases-2 (COX-2) inhibitor [28], NS398; an inhibitor of microglial activation [29], minocycline; a cytokine synthesis inhibitor [30], pentoxifylline (PTX) or a superoxide dismutase (SOD) mimetic [31], tempol was carried out for 14 days. Control infusion of artificial CSF (aCSF) served as the volume and vehicle control.

### Microinjection of test agents into RVLM

Microinjection bilaterally of test agents into RVLM was carried out according to procedures described previously [18-20,24,25] in animals maintained under propofol anesthesia (20 mg/kg/h, Zeneca, Macclesfield, UK). Microinjection was carried out with a glass micropipette (external tip diameter 50 to 80 μm) connected to a 0.5-μL Hamilton microsyringe (Reno, NV, USA). The stereotaxic coordinates for RVLM were: 4.5 to 5.0 mm posterior to lambda, 1.8 to 2.1 mm lateral to midline, and 8.0 to 8.5 mm below dorsal surface of cerebellum. These coordinates were selected to cover the extent of ventrolateral medulla in which functionally identified sympathetic premotor neurons reside. As a routine, a total volume of 50 nL was delivered over 2 to 3 min to allow for complete diffusion of the test agents. Accuracy of the microinjection was confirmed by histological examination of the injection sites. The chemicals used included a superoxide dismutase mimetic, tempol. Microinjection of aCSF served as volume and vehicle control.

### Statistical analysis

Data are expressed as means ± SEM. The statistical software SigmaStat (SPSS, Chicago, IL, USA) was used for data analysis. One-way or two-way analysis of variance with repeated measures was used, as appropriate, to assess group means, to be followed by the Scheffé multiple-range test for *post hoc* assessment of individual means. A *P* value <0.05 was considered statistically significant.

## Results

### Chronic systemic infusion of LPS induces hypertension

**Figure 1:**
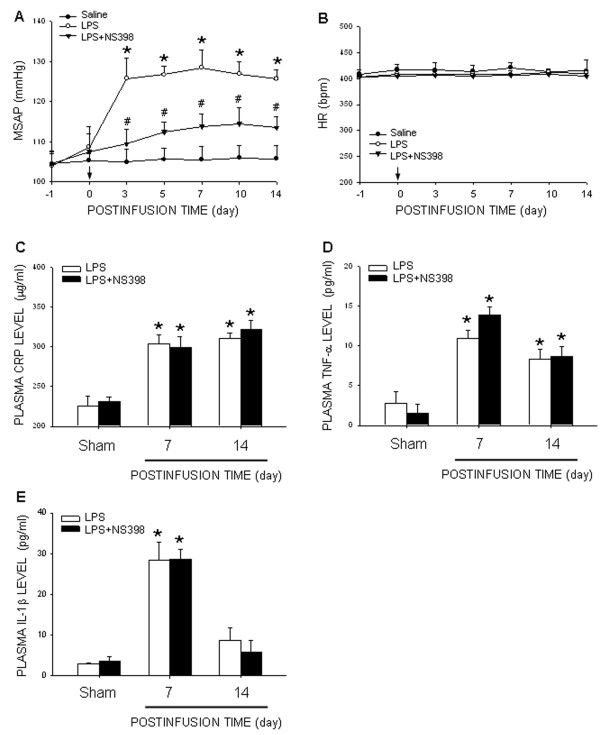
**Infusion of LPS into the peritoneal cavity elevates blood pressure and plasma level of pro-inflammatory cytokines.** Time-course changes in mean SAP (MSAP) **(A)**, HR **(B)**, or plasma level of CRP **(C)**, TNF-α **(D)**, and IL-1β **(E)**, measured on days 7 or 14 after intraperitoneal infusion of saline or LPS (1.2 mg/kg/day) via an osmotic minipump for 14 days, alone or with additional intracisternal infusion of NS398 (1.5 nmol/μL/h). Values are mean ± SEM (*n*=8 to 10 animals in each experimental group). *P <0.05 *vs.* saline-treatment group at corresponding time-intervals or sham-control group; ^#^*P <*0.05 *vs.* LPS-treatment group at corresponding time-intervals in the *post hoc* Scheffé multiple-range test. Arrow indicates the time point during which the osmotic minipump was implanted into the peritoneal cavity.

Compared to saline, infusion of LPS (1.2 mg/kg/day) into the peritoneal cavity by an osmotic minipump induced an increase in mean SAP (MSAP) that became significant on day 3 and lasted for at least 14 days postinfusion (Figure 1A). Of note was the LPS-induced hypertension was significantly inhibited by concurrent IC infusion of a COX-2 inhibitor, NS398 (1.5 nmol/μL/h). However, IP infusion of LPS, alone or with additional IC infusion of NS398, had no significant effect on HR (Figure 1B). Since the LPS-induced hypertension was sustained at a comparable level, subsequent biochemical analyses were carried out at day 7 and/or day 14 to reduce the number of animals used. On day 14 after IP infusion of the endotoxin, there is no significant change in body weight (266±3 *vs.* 260±6 g, *P >*0.05, *n*=16), body temperature (36±1 *vs.* 37±2°C, *P >*0.05, *n*=16), daily food (22±5 *vs.* 23±5 g/day, *P >*0.05, *n*=16), or water (47±5 *vs.* 50±7 mL/day, *P >*0.05, *n*=16) intake between the saline- and LPS-treated animals.

### Chronic systemic infusion of LPS induces inflammatory response

Hypertension induced by IP infusion of LPS was accompanied by an increase in plasma levels of CRP, TNFα, and IL-1β, detected on day 7 or 14 after infusion of the endotoxin (Figure 1C to 1E). However, the LPS-induced systemic inflammatory response was not significantly affected by infusion of NS398 (1.5 nmol/μL/h) into the cisterna magna.

### Chronic systemic LPS infusion upregulates COX-2-dependent expression of pro-inflammatory cytokines in RVLM

**Figure 2:**
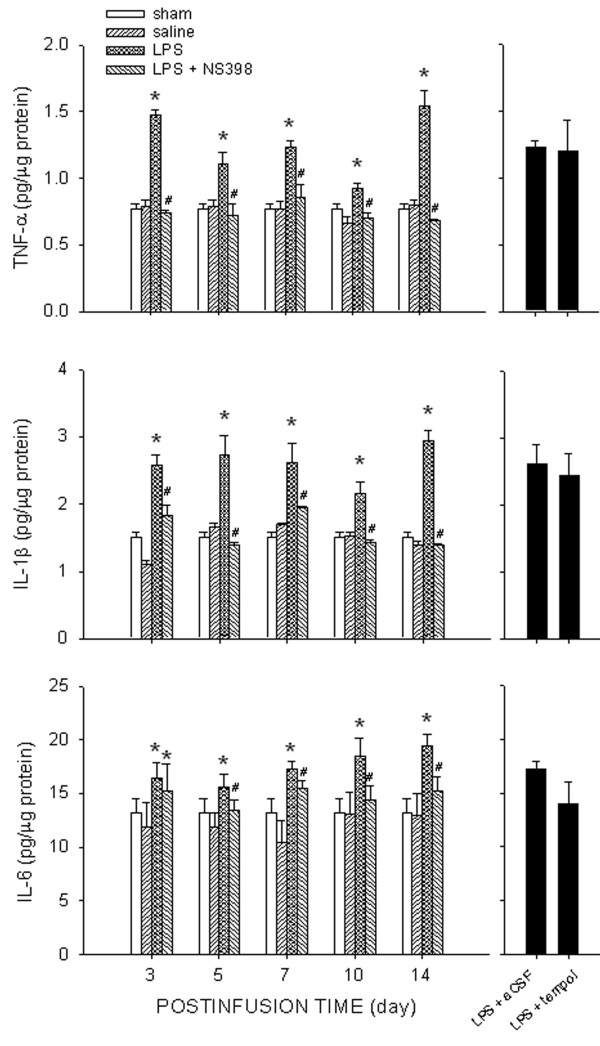
**Intraperitoneal infusion of LPS also elevates tissue level of pro-inflammatory cytokines in RVLM.** Left panels: Time-course changes in tissue level of TNF-α, IL-1β, or IL-6 in RVLM after intraperitoneal infusion of saline or LPS (1.2 mg/kg/day), alone or with additional intracisternal infusion of NS398 (1.5 nmol/μL/h). Right panels: Tissue level of the proinflammatory cytokines in RVLM measured on day 7 after intraperitoneal LPS infusion, with additional intracisternal infusion of tempol (1 μmol/μL/h) or aCSF. Values are mean ± SEM (*n*=8 to 10 animals in each experimental group). *P <0.05 *vs.* corresponding saline-treatment group; ^#^*P <*0.05 *vs.* corresponding LPS-treatment group in the *post hoc* Scheffé multiple-range test.

IP infusion of LPS for 14 days also promoted a significant increase in tissue levels of TNF-α, IL-1β, or IL-6 in RVLM (Figure 2) that became significant on day 3 and lasted for at least 14 days. Whereas concurrent IC infusion of NS398 (1.5 nmol/μL/h) significantly blunted the upregulation of proinflammatory cytokines in RVLM, IC infusion of a SOD mimetic, tempol (1 μmol/μL/h) exerted no discernible effect on the elevated TNF-α, IL-1β, or IL-6 when determined on day 7 after the induction of systemic inflammation. Basal levels of pro-inflammatory cytokines in RVLM were comparable among the experimental groups (data not shown).

### Microglial activation and COX-2 upregulation in RVLM following chronic systemic LPS infusion

**Figure 3:**
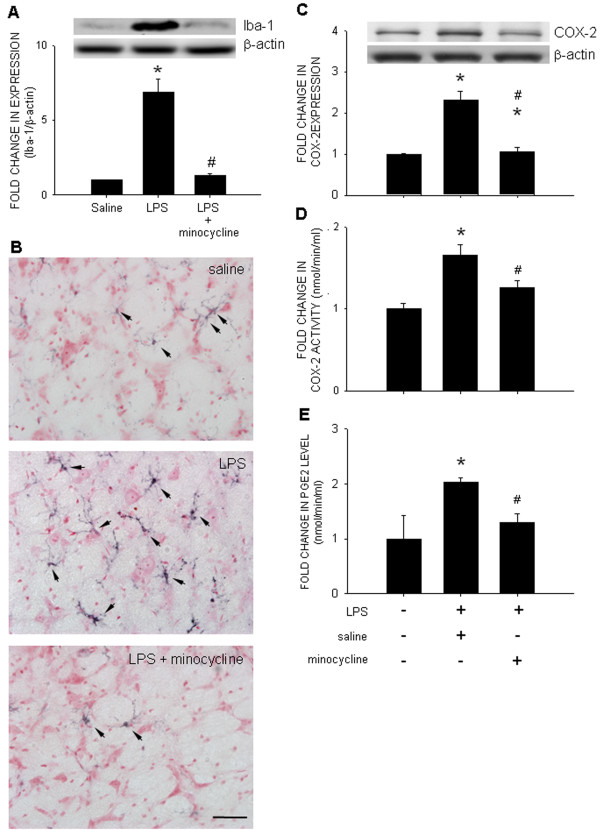
**Systemic inflammation activates microglia and enhances COX-2 activity in RVLM.** Gels (inset) and densitometric analysis of results from Western blot showing changes in expression of Iba-1 **(A)** or COX-2 **(C)**, or representative photomicrographs showing immunoreactivity to Iba-1 (black color) **(B)**, COX-2 activity **(D)**, or tissue level of PGE2 **(E)** in RVLM, examined on day 7 after intraperitoneal infusion of saline or LPS (1.2 mg/kg/day), alone or with additional intracisternal infusion of minocycline (9 nmol/μL/). Values are mean ± SEM (*n*=8 to 10 animals in each experimental group). *P <0.05 *vs.* saline-treatment group; ^#^*P <*0.05 *vs.* LPS-treatment group in the *post hoc* Scheffé multiple-range test. Scale bar in (A): 25 μm.

Compared to saline infusion, the expression of Iba-1 protein, an experimental index for activated microglia [32], and distribution of Iba-1 immunoreactivity in RVLM were markedly increased when measured on day 3 or 7 (data not shown) following IP infusion of LPS; and was antagonized by IC infusion of minocycline (9 nmol/μL/h), an inhibitor of microglial activation (Figure 3A and B). Systemic LPS infusion also increased COX-2 expression and activity, alongside production of PGE2, which were blunted by concurrent IC infusion of minocycline (Figure 3C to E). Treatment with minocycline also significantly antagonized the increase in tissue levels of TNF-α (1.69±0.09 *vs.* 0.74±0.04 pg/μg protein, *P*<0.05, *n*=12) or IL-1β (16.77±1.67 *vs*. 12.13±1.10 pg/μg protein, *P*<0.05, *n*=12) in RVLM induced by systemic LPS infusion. The same agent when infused into the peritoneal cavity had no significant effect on the increased expression of TNF-α (1.69±0.09 *vs*. 1.77±0.07 pg/μg protein, *P*>0.05, *n*=6) or IL-1β (16.77±1.67 *vs*. 16.40±1.74 pg/μg protein, *P*>0.05, *n*=12) in RVLM measured on day 3 following systemic LPS infusion.

### Endothelial dysfunction in RVLM following chronic systemic LPS infusion

**Figure 4:**
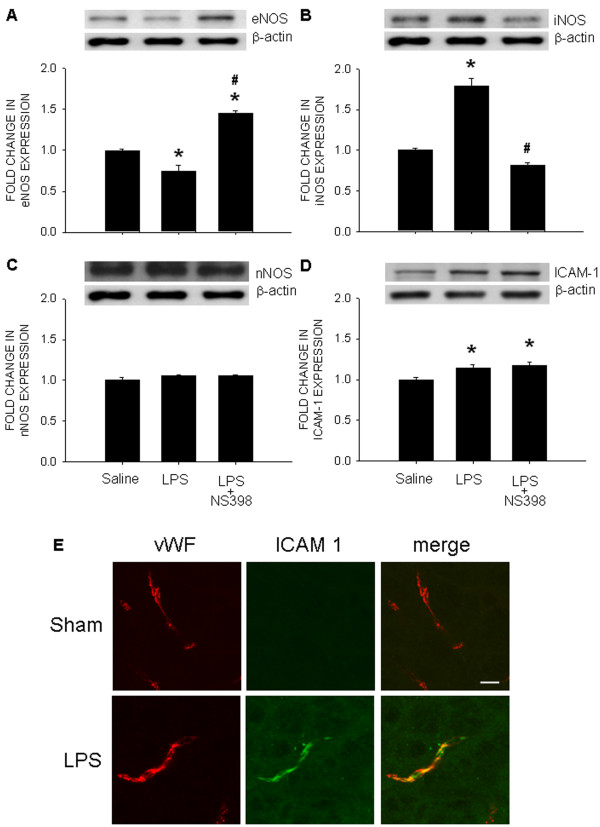
**Intraperitoneal infusion of LPS induces endothelial dysfunction in RVLM.** Representative gels (inset) and densitometric analysis of results from Western blot showing changes in expression of eNOS **(A)**, iNOS **(B)**, nNOS **(C)**, or ICAM-1 **(D)**, as well as photomicrographs showing distribution of vWF- (red fluorescence) and ICAM-1-immunoreactivity (green fluorescence) in RVLM, examined on day 7 after intraperitoneal infusion of saline or LPS (1.2 mg/kg/day), alone or with additional intracisternal infusion of NS398 (1.5 nmol/μL/h). Values are mean ± SEM (*n*=8 to 10 animals in each experimental group). *P <0.05 *vs.* saline-treatment group; ^#^*P <*0.05 *vs.* LPS-treatment group in the *post hoc* Scheffé multiple-range test. Note that colocalization of vWF- and ICAM-1-immunoreactivity is shown in yellow color in (D). Scale bar in (D): 10 μm.

Systemic LPS infusion for 14 days significantly reduced endothelial nitric oxide synthase (eNOS) (Figure 4A) but augmented inducible NOS (iNOS) (Figure 4B) protein expression in RVLM, determined on day 7 or 14 (data not shown) postinfusion. IC infusion of NS398 (1.5 nmol/μL^/^h) antagonized the elevated iNOS expression and reverted eNOS expression from a reduction to an augmentation. However, no apparent change in neuronal NOS (nNOS) expression in RVLM was detected in rats that received peripheral LPS infusion, which was similarly unaffected by NS398 (Figure 4C). Systemic infusion of LPS also increased ICAM-1 expression in RVLM, which was not antagonized by IC infusion NS398 (Figure 4D). Viewed under confocal microscopy, ICAM-1-immunoreactivity was found to co-localize with vWF-immunoreactivity in blood vessels (Figure 4E). LPS, however, did not affect the integrity of the blood–brain barrier, as determined by a maintained extravasation of Evans Blue dye in RVLM tissue (saline: 10.6±3.5 *vs.* LPS: 13.8±4.3 μg/g tissue, *P >*0.05, *n*=8).

### Microglial activation and COX-2 upregulation in RVLM underpin hypertension induced by chronic systemic LPS infusion

The pressor response detected on day 14 after IP infusion of LPS was significantly attenuated by IC infusion of minocycline (9 nmol/μL/h) (145±5 *vs.* 111±5 mmHg, *P <*0.05, *n*=12) or a cytokine synthesis inhibitor, PTX (30 nmol/μL/h) (145±5 *vs.* 115±6 mmHg, *P <*0.05, *n*=12). Infusion into the peritoneal cavity of the same test agents, on the other hand, was ineffective to affect LPS-induced hypertension measured on day 14 postinfusion (minocylcine: 127±3.1 *vs.* 132±4.0 mmHg, *P >*0.05, *n*=8; PTX: 127±3.1 *vs.* 123±3.8 mmHg, *P >*0.05, *n*=8).

### Increase in superoxide production in RVLM following chronic systemic LPS infusion

**Figure 5:**
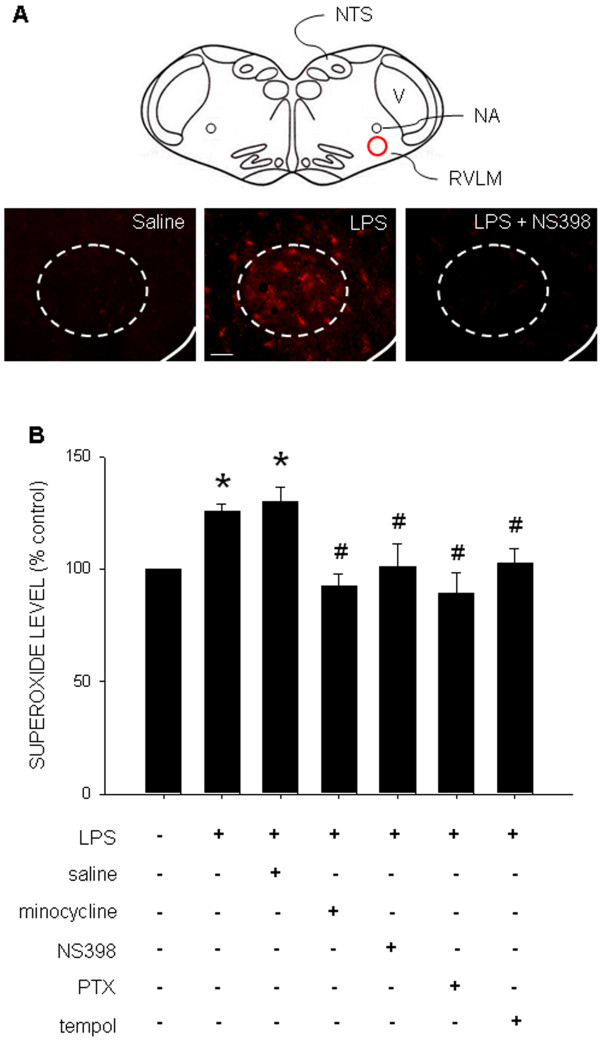
**Intraperitoneal infusion of LPS induces neuroinflammation-associated oxidative stress in RVLM.** Representative photomicrographs showing the distribution of dihydroethidium (red fluorescence) **(A)**, or changes in tissue level of superoxide **(B)** in RVLM examined on day 7 after intraperitoneal infusion of saline or LPS (1.2 mg/kg/day), alone or with additional intracisternal infusion of minocycline (9 nmol/μL/h), pentoxifylline (PTX, 30 nmol/μL/h), NS398 (1.5 nmol/μL/h), or tempol (1 μmol/μL/h). Values are mean ± SEM (*n*=8 to 10 animals in each experimental group). *P <0.05 *vs.* saline-treatment group; ^#^*P <*0.05 *vs.* LPS-treatment group in the *post hoc* Scheffé multiple-range test. A schematic drawing of rostral medulla oblongata is included in (A) to illustrate the location of RVLM from where the photomicrographs were taken. NA, nucleus ambiguous; NTS, nucleus tractus solitarii; V, nucleus spinalis trigemini. Scale bar in (A): 100 μm.

The manifestation of DHE-positive cells and tissue level of O_2_^·-^ in RVLM, measured on day 7 after IP infusion of LPS (1.2 mg/kg/day), were significantly increased (Figure 5). This induced increase in O_2_^·-^ production in RVLM was abolished by IC infusion of NS398 (1.5 nmol/μL/h) (Figures 5A and B), minocycline (9 nmol/μL/h), PTX (30 nmol/μL/h) (Figure 5B), or tempol (1 μmol/μL/h) (Figure 5B).

### Upregulation of the NADPH oxidase subunits and antioxidants expression in RVLM following chronic systemic LPS infusion

**Figure 6:**
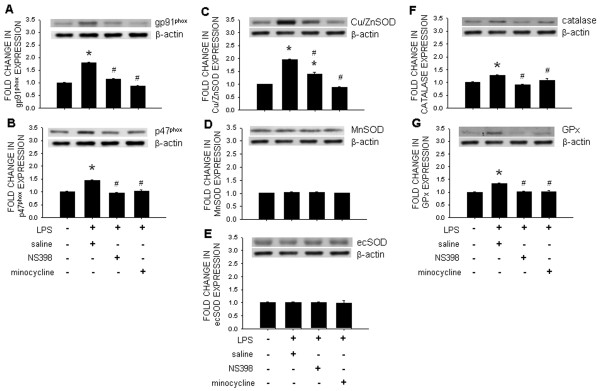
**Intraperitoneal infusion of LPS upregulates NADPH oxidase subunits and antioxidants in RVLM.** Representative gels (inset) and densitometric analysis of results from Western blot showing changes in expression of gp91^phox^ (**A**), p47^phox^ (**B**), Cu/ZnSOD (**C**), MnSOD (**D**), ecSOD (**E**), catalase (**F**), or glutathione peroxide (GPx) (**G**) in RVLM, determined on day 7 after intraperitoneal infusion of saline or LPS (1.2 mg/kg/day), alone or with additional intracisternal infusion of NS398 (1.5 nmol/μL/h) or minocycline (9 nmol/μL/h). Values are mean ± SEM (*n*=8 to 10 animals in each experimental group). *P <0.05 *vs.* saline-treatment group; ^#^*P <*0.05 *vs.* LPS-treatment group in the *post hoc* Scheffé multiple-range test.

IP infusion of LPS for 14 days resulted in a significant increase in the expression of gp91^phox^ and p47^phox^ subunits of NADPH oxidase, which were detected on day 5 (data not shown) and peaked on day 7 (Figure 6A and B) postinfusion. This upregulation was antagonized by IC infusion of NS398 (1.5 nmol/μL/h) or minocycline (9 nmol/μL/h). Systemic infusion of LPS also upregulated the expression of Cu/ZnSOD (Figure 6C), catalase (Figure 6F), or GPx (Figure 6G) in RVLM, but not MnSOD (Figure 6D) or ecSOD (Figure 6E), detected on day 7 and 14 (data not shown) postinfusion. The augmented expression of the antioxidant was blunted by IC infusion of NS398 (1.5 nmol/μL/h) or minocycline (9 nmol/μL/h).

### Oxidative stress in RVLM underpins hypertension induced by chronic systemic infusion of LPS

**Figure 7:**
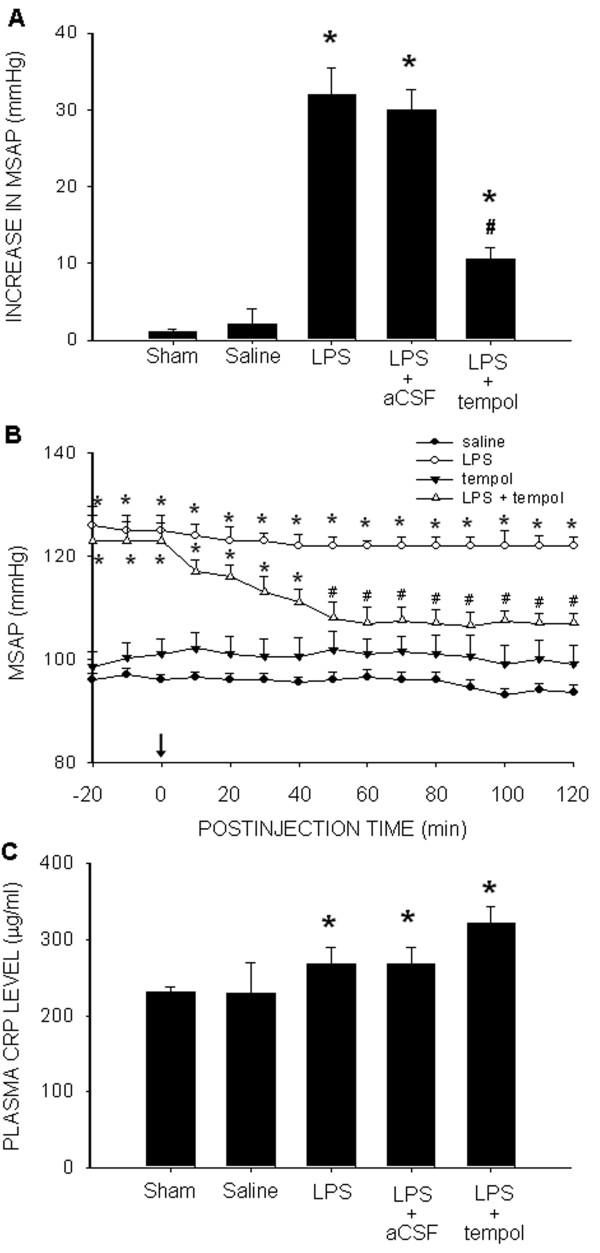
**Oxidative stress in RVLM underpins hypertension induced by intraperitoneal infusion of LPS.** Changes in MSAP **(A,B)** or plasma level of CRP **(C)** determined on day 7 after intraperitoneal infusion of saline or LPS (1.2 mg/kg/day), alone or with additional intracisternal infusion of aCSF or tempol (1μmol/μL/h) (A,C), or microinjection bilaterally into RVLM of tempol (100 pmol) (B). Values are mean ± SEM (*n*=8 to 10 animals in each experimental group). *P <0.05 *vs.* saline-treatment or sham-control group; ^#^*P <*0.05 *vs.* LPS-treatment group in the *post hoc* Scheffé multiple-range test.

IC infusion of tempol (1 μmol/μL/h) significantly attenuated the pressor response to IP infusion of LPS (1.2 mg/kg/day) (Figure 7A). Comparable results were observed acutely for at least 120 min following microinjection of tempol (100 pmol) into the bilateral RVLM (Figure 7B), but not intravenous administration (125±2.1 *vs.* 130±2.8 mmHg, *P >*0.05, *n*=8) at the same dose. IC infusion of tempol, on the other hand, had no appreciable effect on the increase in plasma CRP following LPS infusion (Figure 7C).

### Redox-sensitive downregulation of Kv4.3 potassium channel in RVLM following chronic systemic infusion of LPS

**Figure 8:**
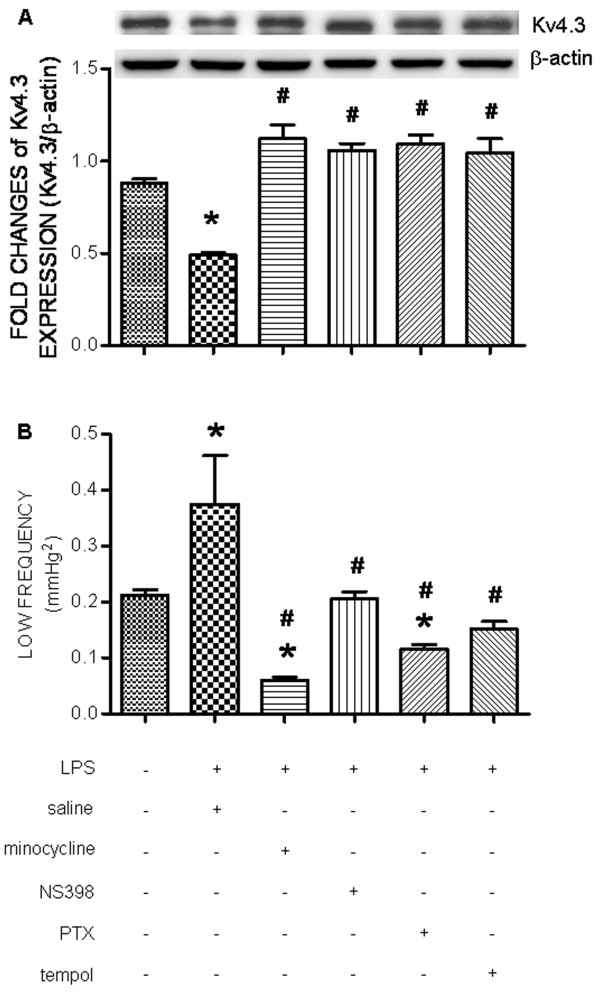
**Intraperitoneal infusion of LPS downregulates Kv4.3 potassium channel in RVLM and increase the sympathetic neurogenic vasomotor activity.** Representative gels (inset) and densitometric analysis of results from Western blot showing changes in expression of Kv4.3 channel protein in RVLM **(A)** or changes in the power density of the low frequency component of SAP spectrum **(B)**, measured on day 7 after intraperitoneal infusion of saline or LPS (1.2 mg/kg/day), alone or with additional intracisternal infusion of minocycline (9 nmol/μL/h), NS398 (1.5 nmol/μL/h), PTX (30 nmol/μL/h), or tempol (1 μmol/μL/h). Values are mean ± SEM (*n*=8 to 10 animals in each experimental group). *P <0.05 *vs.* saline-treatment group; ^#^*P <*0.05 *vs.* LPS-treatment group in the *post hoc* Scheffé multiple-range test

IP infusion of LPS (1.2 mg/kg/day) significantly downregulated the protein expression of the voltage-gated potassium channel, Kv4.3, in RVLM (Figure 8A). This induced downregulation of Kv4.3 was attenuated in animals that received IC infusion of minocycline (9 nmol/μL/h), NS398 (1.5 nmol/μL/h), PTX (30 nmol/μL/h), or tempol (1 μmol/μL/h).

### Neuroinflammation- and oxidative stress-associated increase in neurogenic sympathetic vasomotor activity following chronic systemic infusion of LPS

IP infusion of LPS (1.2 mg/kg/day) significantly increased the power density of the LF component of SAP signal (Figure 8B). This induced increase in our experimental index of neurogenic sympathetic vasomotor activity [22] was attenuated in animals that received IC infusion of minocycline (9 nmol/μL/h), NS398 (1.5 nmol/μL/h), PTX (30 nmol/μL/h), or tempol (1 μmol/μL/h).

## Discussion

Chronic systemic inflammation contributes to the pathogenesis of hypertension. The findings of the present study suggest that neuroinflammation and oxidative stress in RVLM play an active role in this process. Our results showed that systemic inflammation causes endothelial dysfunction and activates microglia in RVLM to induce COX-2-dependent neuroinflammation that leads to an increase in O_2_^·-^ production, contributing to an increase in sympathetic neurogenic vasomotor tone and neurogenic hypertension possibly via downregulation of Kv4.3 potassium channel expression.

IP infusion of gram-negative bacterial endotoxin LPS is a well-characterized rodent model of systemic inflammation. In contrast to high-dose endotoxin that induces a robust yet transient inflammatory response, low-dose endotoxin causes low-grade yet persistent inflammatory responses from the host, as reflected in the mildly sustained levels of inflammatory mediators [33,34]. Indeed, IP infusion of *Escherichia coli* LPS at the dose used in the present study resulted in a significant increase in plasma level of pro-inflammatory cytokine and a moderate increase in plasma level of CRP that is comparable to low-grade systemic inflammation elicited by periodontal bacteria [35] or hyperlipidic diet [36]. CRP is a marker for systemic inflammation [37] and is a risk predictor for symptomatic peripheral arterial diseases [38]. In an earlier clinical study [38], development of vascular diseases and hypertension is more prevalent in participants with an approximately 35% increase in plasma CRP. With a comparable increase in plasma CRP of 27±5% on IP infusion of LPS, our animal model should suitably predict augmented risks of the rats in developing hypertension. Recent clinical studies [39-41] further indicate that low-grade systemic inflammation is associated with a variety of human chronic cardiovascular diseases, including atherosclerosis, diabetes, heart failure, and hypertension.

A major finding in the present study is that the LPS-induced systemic inflammation is associated with neuroinflammation in RVLM. In particular, the temporal profile of the augmented levels of TNF-α, IL-1β, IL-6, or iNOS in RVLM after IP infusion of LPS paralleled that of the elevated plasma pro-inflammatory cytokines and CRP levels. Since most of the blood-borne inflammatory cytokines have restricted access to the brain due to poor passage through the blood-brain barrier [42] and our present finding of an intact blood-brain barrier following systemic LPS infusion, it is conceivable that the observed neuroinflammation in RVLM may not result from direct entry of blood-borne inflammatory cytokines to this neural substrate. Systemic administration of LPS has been demonstrated to induce an innate immune response in brain [14,43]. Thus, it is of interest to find in the present study that microglia was activated in RVLM and the increase in protein expression and enzyme activity of COX-2 was prevented by an inhibition of microglial activation. Furthermore, inhibition of COX-2 ameliorated the augmented expression of the proinflammatory cytokines and iNOS in RVLM. These results therefore complement previous reports [8,44], and suggest that microglial activation and COX-2-dependent mechanism mediate the transfer of systemic inflammation to neuroinflammation in RVLM. The ineffectiveness of IC infusion of NS398 to inhibit peripheral inflammation deems unlikely the antagonism of neuroinflammation in RVLM by the COX-2 inhibitor is secondary to leakage to systemic circulation. PGE2, the eicosanoid product of COX, was reported to mediate the initiation and development of inflammation after entering the brain [15,45]. A reversal by minocycline and NS398 on PGE2 production in RVLM implies the involvement of COX-PGE2 signaling in mediating neuroinflammation in RVLM following systemic inflammation. It is noteworthy that the expression of proinflammatory cytokines detected in RVLM in this model of LPS-induced neurogenic hypertension is comparable to that found in the brain of other animal models of hypertension with neurogenic components, including chronic infusion of angiotensin II [7,14,46] and spontaneously hypertensive rats [5,10,12]. Together, they highlight the importance of neuroinflammation in the manifestation of neurogenic hypertension in a variety of animal model of hypertension.

The endothelium effectively maintains an anti-inflammatory environment under normal conditions [47]. In addition, a loss of endothelial integrity and an increase in expression of adhesion molecules are important attributes to tissue damage during inflammation [48]. We showed in the present study that endothelial dysfunction, characterized by a downregulation of eNOS and an upregulation of ICAM-1 in blood vessels occurred in RVLM following chronic systemic inflammation. The observation that IC infusion of a COX-2 inhibitor exerted minimal effect on ICAM-1 upregulation in RVLM suggests that COX-2 activation is downstream to the loss of endothelial integrity in RVLM. That IC infusion of NS398 reverted eNOS expression from a reduction to an augmentation further suggested the presence of a rebound compensatory mechanism.

We reported previously [24,49] that generation of O_2_^·-^ in RVLM because of an upregulation of gp91^phox^ or p47^phox^ subunit of NADPH oxidase underlies neurogenic hypertension. The present study further showed that this oxidative stress elicited by systemic inflammation is downstream to microglial activation via a COX-2-dependent mechanism. These results are parallel to a recent report [50] on the production of O_2_^·-^ in the PVN to neuroinflammation induced by intracerebroventricular administration of LPS. Mechanisms involved in activation of NADPH oxidase in RVLM under neuroinflammation is not immediately clear. In this regard, the enzyme activity of Rac, a member of the Rho family small GTPases that plays a crucial role in activation of NADPH oxidases [51], was reported to be activated by microglia-specific protein Iba-1 under inflammation condition [52]. Based on the temporal expression profiles, our results showed that upregulation of gp91^phox^ or p47^phox^ subunit of NADPH oxidase after systemic inflammation precedes that of the increases in Cu/ZnSOD, catalase, or GPx in RVLM. The antagonism by IC infusion of NS398 or tempol on upregulation of the antioxidants may represent yet another cellular compensatory response to cope with oxidative stress induced by neuroinflammation in RVLM. Our findings of a sustained increase in O_2_^·-^ despite upregulation of antioxidants further suggest that the production of O_2_^·-^ in RVLM may exceed its degradation under neuroinflammation. Alternatively, it may result from inhibition of enzyme activities of the antioxidants by neuroinflammation. A lack of change in MnSOD expression in RVLM may imply a differential involvement of cytosolic versus mitochondrial antioxidant in this compensatory response under neuroinflammation.

Results from our physiological evaluations provided the crucial documentation that oxidative stress induced by COX-2-dependent neuroinflammation in RVLM underpins the hypertension elicited by chronic systemic inflammation. We found that the progression of hypertension coincided temporally with the appearance of neuroinflammation and oxidative stress in RVLM after IP infusion of LPS, which was similarly antagonized by IC, but not systemic infusion of minocycline, NS398, PTX or tempol. The suppression of LPS-induced increase in sympathetic neurogenic vasomotor activity by the same treatments further indicates that the oxidative stress-associated neurogenic hypertension in response to neuroinflammation in RVLM is mediated via an increase in sympathetic outflow to the blood vessels. Since microinjection of tempol into RVLM only partially blunted the LPS-induced hypertension, the contribution by oxidative stress in other brain regions cannot be ruled out. In this regard, oxidative stress in PVN [50,53] and nucleus tractus solitarii [12,54] contributes to the pathogenesis of neurogenic hypertension, and neuroinflammation occurs in both brain regions [4,8,12,50] in response to systemic inflammation. In addition, a recent study [55] reported that COX-1-derived PGE2 signaling in the subfornical organ is required for ROS-mediated neurogenic hypertension induced by systemic infusion of angiotensin II.

The A-type voltage-gated potassium (Kv) channels play pivotal roles in regulating the intrinsic excitability and the firing properties of neurons. The functional diversity of neuronal Kv currents is generated, in part, through the expression of multiple Kv channel pore-forming (α) subunits [56]. We found in the present study that neuroinflammation in RVLM following systemic LPS infusion was associated with a redox-sensitive reduction in the expression of Kv4.3 channel protein because of microglial activation and the presence of COX-2 and cytokine. Kv4.3 contributes to the transient outward potassium current; and its activation leads to reduction in neuronal excitability by increasing the duration of action potential [57]. By reducing Kv4.3 channel protein expression, it is thus reasonable to speculate that neuroinflammation in RVLM promotes hypertension via sympathoexcitation that may result from a redox-sensitive increase in neuronal excitability. In RVLM, redox-sensitive downregulation of Kv4.3 potassium channel was reported [58] to mediate sympathoexcitation in heart failure. We noted that apart from Kv4.3, Kv1.4 and Kv4.2 are also sensitive to tissue inflammation [59]. The contribution of these pore-forming subunits of potassium channel in RVLM in redox-sensitive sympathoexcitation under neuroinflammation, however, awaits further delineation. It is also noteworthy that whereas nNOS-derived NO in RVLM mediates pressor response via activation of sympathetic vasomotor activity [60], the minimal alteration in nNOS expression during chronic systemic inflammation suggests that this signaling pathway plays a minor role in the pathogenesis of neurogenic hypertension during chronic systemic inflammation.

Hypertension alone activates circulating T-cells to develop vascular inflammation [7]. Moreover, LPS produces a robust inflammatory reaction in brain, which is significantly diminished when the pressor responses is normalized with angiotensin II receptor antagonist [14]. It thus is possible that neuroinflammation in RVLM may be secondary to systemic hypertension induced by IP infusion of the endotoxin. Our preliminary results indicated that this possibility is not operable. Oral intake for 7 days of amlodipine, a calcium channel inhibitor that normalizes hypertension with minimal central actions [61], promoted a decrease in blood pressure with minimal effect on the increases in pro-inflammatory cytokine expression or O_2_^·-^ production in RVLM after chronic systemic inflammation. The possibility that blood-borne, instead of cytokines produced in RVLM underlies the observed hypertension after IP infusion of LPS is also deemed minimal. IC infusion of NS398, at a dose that reduced pressor responses and inhibited pro-inflammatory cytokines expression in RVLM exerted minimal effect on the increase in plasma TNF-α, IL-1β, or CRP level. Furthermore, IP infusion of same dose of PTX or NS398 was also ineffective against the induced hypertension following systemic inflammation.

## Conclusion

Our results support the novel notion that chronic systemic inflammation causes endothelial dysfunction, activates microglia and induces COX-2-dependent neuroinflammation, followed by oxidative stress in RVLM that leads to the development of neurogenic hypertension possibly via downregulation of the voltage-gated Kv4.3 channel. A feed-forward mechanism for the development of hypertension under chronic inflammation was proposed [7]. According to this proposal, a modest degree of elevation in blood pressure promotes inflammation that further raises blood pressure, leading to severe hypertension. Our results added a new dimension to this proposal by demonstrating that the pathogenesis of hypertension during chronic systemic inflammation can take origin from brain via neuroinflammation-associated oxidative stress in RVLM. We further extended the previous studies [4,8,12,62] to reveal that ROS, in particular O_2_^·-^, may serve as the crucial link between systemic inflammation, neuroinflammation, and neurogenic hypertension.

## Abbreviations

aCSF: Artificial cerebrospinal fluid; COX-2: Cycloxygenase-2; CRP: C-reactive protein; Cu/ZnSOD: Copper/zinc superoxide dismutase; DHE: Dihydroethidium; EB: Evans blue; ecSOD: Extracellular superoxide dismutase; eNOS: Endothelial nitric oxide synthase; GPx: Glutathione peroxidase; HR: Heart rate; ICAM-1: Intercellular adhesion molecule-1, IC, Intracisternal; IL: Interleukin; iNOS: Inducible nitric oxide synthase; IP: Intraperitoneal; Kv: Voltage-gated potassium channel; LF: Low frequency; LPS: Lipopolysaccharide; MnSOD: Manganese superoxide dismutase; MSAP: Mean systemic arterial pressure; nNOS: Neuronal nitric oxide synthase; O_2_^·-^: Superoxide anion; PBS: Phosphate buffered saline; PGE2: Prostaglandin E2; PTX: Pentoxifylline; PVN: Paraventricular nucleus of the hypothalamus; ROS: Reactive oxygen species; RVLM: Rostral ventrolateral medulla; SAP: Systemic arterial pressure; SOD: Superoxide dismutase; TNF-α: Tissue necrosis factor-α; vWF: Von Willebrand factor.

## Competing interests

The authors declare that they have no competing interests.

## Authors’ contributions

KLHW and JYHC conceived and designed the study as well as analyzed and interpreted the data, and wrote the manuscript. KLHW performed experiments for data acquisition and performed the statistical analysis. SHHC was involved in the design of the study, overall data interpretation, and revising the manuscript critically for important intellectual content. KLHW and JYHC share primary responsibility for final content. All authors have read and approved the final version of this manuscript.

## Authors’ information

KLHW is currently a principle investigator in the Center in Translational Research in Biomedical Sciences in Kaohsiung Chang Gung Memorial Hospital. SHHC is the Endowed National Chairprofessor in Neuroscience awarded by the Ministry of Education, Taiwan. He is also the founding and current director of the Center in Translational Research in Biomedical Sciences in Kaohsiung Chang Gung Memorial Hospital. SHHC is currently the President of the Federation of Asian and Pacific Pharmacological Societies. JYHC is the Chairprofessor in the Center in Translational Research in Biomedical Sciences, and Director of Department of Medical Research in Kaohsiung Chang Gung Memorial Hospital. She is currently the President of the Federation of Asian and Pacific Physiological Societies.
